# Potential Mechanism for PM_10_ Effects on Birth Outcomes: *In Utero* Exposure Linked to Mitochondrial DNA Damage

**DOI:** 10.1289/ehp.120-a363b

**Published:** 2012-08-31

**Authors:** Tanya Tillett

**Affiliations:** Tanya Tillett, MA, of Durham, NC, is a staff writer/editor for *EHP*. She has been on the *EHP* staff since 2000 and has represented the journal at national and international conferences.

A number of studies have associated exposure to particulate matter (PM) with adverse birth outcomes such as low birth weight. Much is still unknown, however, about the mechanisms that might induce these outcomes. Researchers now report an association between abnormal placental mitochondrial DNA (mtDNA) content—a marker indicative of mitochondrial dysfunction, which may be related to the development of some diseases—and fetal exposure to coarse PM (PM_10_) during the last trimester of pregnancy **[*EHP* 120(9):1346–1352; Janssen et al.]**. Although similar to nuclear DNA in composition and function, mtDNA has fewer protective components and less-efficient repair mechanisms, rendering it particularly vulnerable to oxidative damage.

The 178 mothers in the study, who had given birth between 5 February 2010 and 3 April 2011, were part of a larger prospective initiative called ENVIR*ON*AGE. The women were classified by age, ethnicity, smoking status, place of residence, and other demographics. Regional background levels of PM_10_ were calculated for each mother’s home address using satellite-based data, and the distance from each home to a major road was geocoded. Mothers’ residential PM_10_ exposures were calculated for the following time periods: 0–7 days before delivery, each trimester of pregnancy (1–13 weeks, 14–28 weeks, and 29 weeks to delivery), and the last month of pregnancy.

Immediately after each delivery the research team collected the placenta and a sample of umbilical cord blood, which were assessed for mtDNA content. They then analyzed the relationship between mtDNA content and PM_10_ exposure as well as residential distance to major roads. They found that a 10-µg/m^3^ increase in PM_10_ exposure was associated with a 10.1% reduction in placental mtDNA content in the last week of pregnancy, a 16.1% reduction in the last month of pregnancy, and a 17.4% reduction in the third trimester. Living closer to a major road—an indication of higher exposure to air pollution from traffic—was also associated with lower placental mtDNA content.

No statistically significant association was observed between cord blood mtDNA content and PM_10_ exposure during any defined time period. The researchers speculate this could reflect tissue-level differences in exposure or effects.

The study is limited by the researchers’ inability to exclude the possibility of residual confounding or misclassification, but the findings suggest that prenatal PM_10_ exposure during the last trimester of pregnancy may cause mitochondrial dysfunction, indicating a possible window of susceptibility. Future research is needed to understand the potential health effects of decreased mtDNA content.

